# A Review on the Role of Earthworms in Plastics Degradation: Issues and Challenges

**DOI:** 10.3390/polym14214770

**Published:** 2022-11-07

**Authors:** Shahad Khaldoon, Japareng Lalung, Umrana Maheer, Mohamad Anuar Kamaruddin, Mohd Firdaus Yhaya, Eman S. Alsolami, Hajer S. Alorfi, Mahmoud A. Hussein, Mohd Rafatullah

**Affiliations:** 1 Environmental Technology Division, School of Industrial Technology, Universiti Sains Malaysia, Penang 11800, Malaysia; 2 Green Biopolymer, Coatings & Packaging Cluster, School of Industrial Technology, Universiti Sains Malaysia, Penang 11800, Malaysia; 3 Regional Centre of Expertise, Centre for Global Sustainability Studies, Universiti Sains Malaysia, Penang 11800, Malaysia; 4 School of Dental Sciences, Health Campus, Universiti Sains Malaysia, Kubang Kerian 16150, Malaysia; 5 Chemistry Department, Faculty of Science, King Abdulaziz University, P.O. Box 80203, Jeddah 21589, Saudi Arabia; 6 Chemistry Department, Faculty of Science, Assiut University, Assiut 71516, Egypt

**Keywords:** earthworms, digestive mechanisms, microplastic, degradation, soil ecosystem

## Abstract

Recently, the contribution of earthworms to plastic degradation and their capability to swallow smaller plastic fragments, known as microplastics, has been emphasized. The worm physically changes the size of microplastics and enhances microbial activities to increase the possibility of degradation. However, no research has shown that earthworms can chemically degrade microplastics to an element form, CO_2_ or H_2_O. In this review, previous research has been thoroughly explored to analyse the role that earthworms could play in plastic degradation in the soil. Earthworms can significantly affect the physical characteristics of plastics. However, earthworms’ abilities to chemically degrade or change the chemical structure of plastics and microplastics have not been observed. Additionally, earthworms exhibit selective feeding behaviour, avoiding areas containing a high plastics concentration and rejecting plastics. Consequently, earthworms’ abilities to adapt to the microplastics in soil in the environment can cause a problem. Based on this review, the challenges faced in earthworm application for plastic degradation are mostly expected to be associated with the toxicity and complexity of the plastic material and environmental factors, such as the moisture content of the soil and its temperature, microbial population, and feeding method.

## 1. Introduction

Concern regarding plastics and microplastics (particles smaller than 5 mm) polluting the soil and possibly affecting the biodiversity of the soil biota through the application of commercial fertilizer has been growing [1]. The sewage sludge produced by WWTPs is highly contaminated with microplastics, reaching levels of 90% to 98% kg/kg [1,2]. Furthermore, an additional four billion pieces of microplastic are released from Waste-Water Treatment Plants (WWTPs) facilities to marine environments each year [3]. The cumulative microplastics in the soil can significantly affect its bulk density, microbial activity, water-holding capacity, and water-stable aggregates [4]. Moreover, these microplastics affect the soil biota and biophysical structure. As a result, removing microplastics from the ground is essential to ensure good soil quality. Therefore, plastic pollution in the soil, especially microplastics, has a crucial impact on the food chain and human health. 

Earthworms are the most abundant animal biomass in most global ecosystems [5]. They ingest large amounts of material, of which 2–15% is organic material [6]. They expend a large amount of energy on soil modification. As a result, 74–91% of integrated carbon is respired [7]. An earthworm can alter the soil environment through its ability to impact its physical properties by swallowing huge quantities of soil minerals and organic matter and influencing the soil aggregate stability, porosity, infiltrability, bulk density, and hydraulic conductivity. They play a significant role in pedoturbation [8]. The interaction between earthworms and microorganisms can stimulate the rate of microbiological decomposition of organic matter [9]. It can also increase microbial populations and modify the physicochemical properties of organic material [9,10]. Considering the ability of earthworms to contribute to soil quality, their impact on the soil ecosystem is important [11]. Thus, earthworms have a significant impact on soil structure, are not primarily involved in trophic relationships, and have been identified as typical ecosystem engineers [5,12]. Furthermore, earthworms represent an outstanding potential companion for humans in managing ecosystem services [13]. As a result, they could be an excellent option for assisting in the degradation of plastics in the soil ecosystem. Nevertheless, to utilise earthworms for plastic degradation, it is important to identify the functions earthworms can offer in plastic degradation and the issues and challenges that accompany it. Previously, few attempts have been made to use vermicompost and earthworms to degrade plastic films and microplastics. However, explorations of the potential use of earthworms in microplastic degradation are still lacking. 

**Figure 1:**
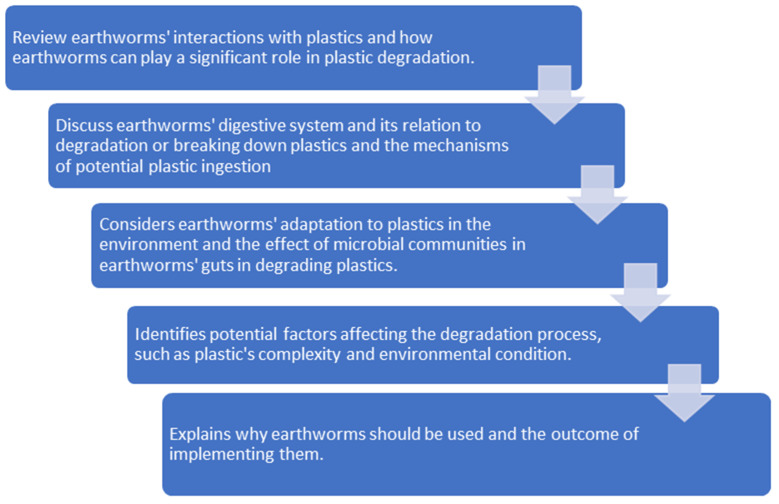
Purpose of this review article.

This review article specifically investigates the possibilities of earthworms being employed for plastic and microplastic degradation, along with the potential disputes and concerns that accompany it. The purpose of this review article is showcased in Figure 1. This review would be beneficial to reveal the potential use of earthworms to degrade plastics and microplastics in the soil and assist in proposing relevant technical approaches for their application.

## 2. Role of Earthworms in Physical and Chemical Degradation of Plastics

There has been conflict regarding the possibility of earthworms to break down or degrade plastics or microplastics. Polymer degradation is defined as any change in physical or chemical properties caused by environmental factors, such as light, heat, and moisture, as well as biological activity [14]. However, few studies have shown the physical changes in the microplastics and ingestion of microplastics due to earthworm activities [15,16,17,18].

Earthworms’ (*Eisenia Foetida* and *Eisenia Andrei*) consumption of five plastic types has resulted in a mass reduction, and polyethene, nylon, polypropylene, ethylene–vinyl acetate, and linear low-density polyethene were among the polymer powders studied [18]. Even though the study found a decrease in polymer mass, it is possible that this was unrelated to earthworm digestion. Instead, it may have indicated the ingestion and accumulation of the microplastics by the earthworms. 

In conclusion, the earthworms’ mass was related to the earthworms’ basal metabolism. Thus, mass reduction of the earthworms’ weight may be related to the microplastics’ interaction with earthworms’ foods, causing a reduction in the food’s organic matter content, resulting in reduced nutrient intake. Furthermore, the earthworms’ weight reduction may also have been a side effect of the interaction between the microplastics and earthworms’ guts, with increased mucus production to protect it from potential damage by microplastics. This reduction is related to earthworms’ defence mechanisms. When inorganic matter is ingested, they tend to produce more mucus in their gut to protect their digestive system from indigestible materials. The gut mucus accelerates organic material decomposition and humification, and may even increase microbial activity, growth, and species diversity in a vermicomposting system [19]. In contrast, Lwanga et al. [20] discovered that, when low-density polyethene microplastics passed through the guts of *Lumbricus terrestris*, their size decreased. However, there was no reduction in plastic mass, indicating that the earthworms were not able to degrade the low-density polyethene microplastics during the feeding period, only affecting their physical properties. Thus, this shows that the digestive tract of earthworms can break down microplastics into smaller sizes; however, there is no degradation. Furthermore, Alauzet et al. [17] discovered that the earthworm *Eisenia Andrei* could consume two lactic-acid-based stereo-copolymers and corresponding oligomer-impregnated paper or coated tree leaves. Although the earthworms were able to consume certain oligomers, the molecular mass of the oligomers had a negative relationship with polylactic acid degradation. The worms could not consume the high-molar-mass PLA polymers directly, regardless of the stereo-copolymer composition. As a result, they could only consume them after the PLA was treated. Even though these plastics were made from biodegradable materials, the complexity of the plastic materials played the key role in determining the degradation process. In previous studies, earthworms have displayed certain abilities to degrade microplastics [15,21]. In addition, further degradation may occur after the excretion of microplastics in their vermicasts through microbial activity [21]. Table 1 below shows the involvement of worms in breaking down microplastics.

From the studies listed in Table 1, it can be summarized that earthworms can ingest microplastics. However, there is not enough evidence of what happens to microplastics after being consumed. Furthermore, the ingestion of certain plastics in lower concentrations caused high earthworm mortality, demonstrating the earthworms’ significant sensitivity. Thus, there are further challenges to overcome regarding the involvement of earthworms in plastics degradation. Nevertheless, according to the early studies, a positive relationship between earthworms and plastic degradation may exist, requiring further investigation.

## 3. Earthworms’ Interactions with Plastics 

### 3.1. The Digestive System of Earthworms and Its Relation to the Degradation or Breakdown of Plastics 

An earthworm’s digestive system consists of a pharynx, oesophagus, and gizzard (called the ‘reception zone’), followed by an anterior intestine that secretes enzymes and a posterior intestine that absorbs nutrients [32,33]. The active muscles and the actions through the digestive portion of an earthworm cause a linking process of the swallowed materials with the enzyme-filled liquid. Blended with the matter by the oesophagus, these digestive liquids expel sugars, amino acids, fungi, bacteria, protozoa, nematodes, etc., as well as decomposed animal and plant materials from the worms’ food. Furthermore, the earthworms’ intestinal membranes work on the degradation of simple remains to be used as an energy source [10]. Thus, a similar effect is expected when microplastics enter earthworms; at the first zone of the body, the microplastics will be exposed to acid mucus, followed by the grinding and mixing in the gut and intestine area. Finally, the microplastics will pass through a peritrophic membrane of intestines, which enclose the undigested materials and cover the casts when ejected. Furthermore, the removed matter from worms is rich in important nutrients that attract microbes, resulting in additional nutrient mineralisation [10]. This shows that earthworms can provide a suitable environment and assist in the biodegradation process of microplastics throughout the digestive system and after ejecting them in the cast.

### 3.2. Worms’ Adaptations to Plastics in the Environment 

Some studies have shown the incapability of earthworms to survive in soils contaminated with certain types of plastics and microplastics at certain concentrations [16,17,22,25,26,27,28,30,34,35]. The mortality rate of earthworms is highly influenced by the plastic material, even at low concentrations. The vermitoxicity of microplastics in terms of earthworm mortality has been studied for biodegradable plastics and non-biodegradable plastics [16,17,22,35]. Polystyrene and low-density polyethene are classified as stage-two hazards, capable of causing irritation and damage to living beings [24]. One study found that the mortality of earthworms increased with increasing concentration of polystyrene microplastics in food when earthworms were exposed to polystyrene microplastics (58 µm) at concentrations of 0, 0.25, 0.5, 1, and 2% *w*/*w* [22]. Similarly, when low-density polyethene microplastics were introduced to earthworms at various concentrations and sizes, it resulted in a mortality of 8 to 26% for the low-density polyethene concentration of 28 to 60% *w*/*w* and a mortality of 20 to 30% for the concentration of 1% *w*/*w* [21,35]. This shows that both polystyrene and low-density polyethene microplastics contributed to the death of the earthworms. Moreover, polystyrene microplastics exhibited higher vermitoxicity and worm growth inhibition at 1 and 2% *w*/*w* concentrations. For low-density polyethene microplastics, the mortality rate was detected at concentrations ranging from 28% to 60% *w*/*w* and 1% *w*/*w* in two separate studies [21,35]. These two materials are classified as category-two environmental hazards, and worm mortality was higher when they were fed with both materials [24]. This classification indicates that, when the two materials are combined to form microplastics and enter the environment, they will be hazardous to the environment’s biotic components. *Eisenia Andrei* and *Lumbricus Terrestris* were poisoned by biodegradable plastics, such as polylactic acid and starch-based bioplastics composed of 37.1% pullulan, 44.6% polyethene terephthalate, and 18.3% polybutylene terephthalate [17,35]. The mortality caused by the toxicity of these bioplastics, on the other hand, was significantly different. For example, polylactic acid had no effect on earthworm mortality, whereas starch-based bioplastics had a mortality rate of 40% to 50%. Thus, not all bioplastics can be considered environmentally friendly compared with synthesised plastics. The type of bioplastic can be a considerable challenge in involving earthworms in their degradation, as it could potentially harm them.

Even though most of the degradation testing of plastics is conducted under controlled conditions, especially temperature, which plays the main role in plastic degradation and impacts earthworms, there is a great difference in the degradation or breakdown of plastic materials in the natural environment. Napper and Thompson [36] investigated the deterioration of plastic bags composed of oxo-biodegradable, biodegradable, compostable, and conventional polymers in the soil, the marine environment, and under laboratory conditions, and reported that oxo-biodegradable and biodegradable plastic packs remained in the soil and aquatic environments for three years with clear intaglio physical properties. The tensile stress, on the other hand, decreased over time. Furthermore, compostable plastic sacks were discovered in soil after 27 months of decomposition, even though their physical properties had been significantly weakened. Thus, the results of standard biodegradation tests, ISO17556, must be interpreted with caution due to differences in the conditions and microbial consortia between the standard test and the environment, which determine the difference in the biodegradation capacity. However, there has not been enough research documenting the interaction between earthworms and microplastics in the environment. 

There is a possibility that earthworms avoid soil contaminated with microplastics or plastics. Karthikeyan et al. [37] found that earthworms rejected or avoided media containing a large amount of sand. In addition, some rejection of microplastics might occur due to their morphological characteristics, which may cause physical damage to earthworms’ bodies, leading to further selective behaviour regarding food or the avoidance of areas containing high amounts of microplastics., Nevertheless, some researchers provided evidence that earthworms can exist in soil containing a certain concentration of microplastics under controlled conditions. Thus, the capability of earthworms to degrade microplastics in the natural soil environment is questionable because all of the microplastic degradation studies were conducted under controlled conditions. It might be difficult for earthworms to coexist and break down several types of plastics, even though they are biodegradable plastics, due to the unfavourable natural environment. This requires an in-depth study of the possibility of earthworms coexisting with microplastics (both biodegradable and non-degradable plastics) and their interaction with the soil ecosystem, taking into account research that indicates that those interactions could be harmful. 

### 3.3. Would There Be Any Negative Outcomes of Using Earthworms?

While the application of earthworms in breaking down plastics can be an attractive option, it may come with some severe negative side effects. Their natural behaviours, such as their ability to burrow and eject their product into various levels of the soil structure, may contribute to the spread of microplastics in the soil structure. Few studies, such as [15,21,29], have investigated the possibility of surface microplastics being carried by earthworms into the soil structure. The transport of microplastics by earthworms has the potential to affect other soil ecosystems and leach into water bodies through earthworm burrows. Various living organisms in the soil ecosystem would also help to move microplastics deeper into the soil. Earthworms could move particles in two ways. First, by adhering to earthworms’ bodies when encountering the pollutant. Second, in earthworms’ vermicasts as a result of ingesting microplastics.

When compared with the surface area, *L. terrestris* burrows contained a higher concentration of microplastics. The concentration of microplastics in the burrows rose by up to 25% [16]. De Souza Machado et al. [38] found that microplastics in the soil might have consequence for biodiversity and agro-ecosystems. This demonstrates that earthworms can consume microplastics and eject them as unconsumable material into the environment, thereby providing more pathways for microplastics to enter the soil profile and groundwater. Furthermore, they may even contribute to microplastic (100 nm) absorption by the plant’s roots [39], as earthworms may grind microplastics to a smaller size, making them absorbable by the roots. Microplastics can release and absorb pollutants taken up by plants’ roots and affect their health. Thus, this factor needs to be considered when conducting future studies on the implementation of earthworms for plastic deterioration.

## 4. The Challenges of Implementing Earthworms as an Agent of Plastic Degradation 

### 4.1. Plastics Rejection

Zhang et al. (2018, [23]) reported that earthworms (*Lumbricus terrestris*) had different interactions with diverse types of plastics. *Lumbricus terrestris* had selective behaviour when provided with food mixed with polyethene mulch material, even though it was buried in the soil. Similar behaviour was observed with field-weathered biodegradable plastic mulches. This shows that earthworms tend to avoid plastic material, regardless of whether it is bio-degradable or not. However, when biodegradable plastics underwent composting and soil burial, some of them were fully eaten and could not be recovered. Thus, the condition that the material is in also seems to play a major role in attracting worms to the plastic material and ingesting it. For this, the common practice of force-feeding earthworms with microplastics may lead to the false interpretation of the earthworms’ capability to take in plastic materials when they are in their ecosystem. The smell of polymer monomers may also attract earthworms and lead them to consume them [31]. This illustrates how sensitive earthworms are to their environment, thus requiring further understanding. 

### 4.2. Plastic Complexity

The complexity of plastic materials, which includes their shapes, sizes, and compositions, is another important factor influencing the degradation process [40]. Most available plastics used in industry are composed of multiple polymers, blends, or low-molecular-weight additives, rather than a single chemical homogeneous component (e.g., plasticisers). Moreover, various structural co-polymer elements can be present within one polymer itself. These may be scattered statistically along the random co-polymer chains, distributed alternately, or used to build longer blocks of each structure. Another structural characteristic of a polymer is the possibility of chain branching or the formation of cross-linked polymer networks. Despite having the same overall composition, the different structures of a polymer can have a direct impact on the material’s accessibility to enzyme-catalysed polymer chain cleavage [41]. This difference also has a significant impact on higher-ordered polymer structures, such as crystallinity and the glass transition temperature, which have been shown to control the degradation pattern of many polymers [42]. Furthermore, crystallinity and crystal morphology are affected by the processing procedure and can change over time. Thus, degradation is highly influenced by the complexity of plastic materials. 

Regardless of the environmental factor, the material itself has an impact on its degradation. Material complexity has a significant impact on microbial attraction [42]. 

**Figure 2:**
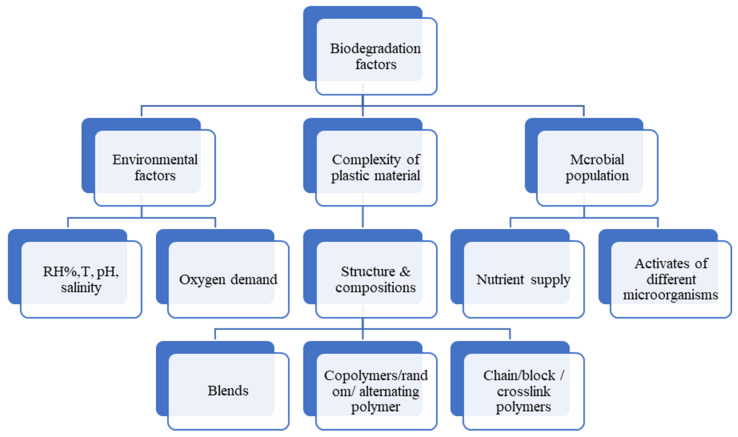
Environmental factors that influence biodegradation.

In conclusion, the factors that affect others are interrelated, as shown in Figure 2. If the environmental conditions are favourable, biodegradation is likely to occur for certain types of polymers.

### 4.3. Microbial Activity and Environmental Conditions

Plastics are well-known for their tensile strength and resistance to water [43]. As a result, the biodegradation process is not entirely efficient in the natural environment. Certain factors, such as the microbes’ populations and their activities, must be taken into account for biodegradation to take place. Moisture content, pH, temperature, salinity, the absence or presence of oxygen, and the availability of various nutrients all have significant impacts on microbial activity, affecting polymer decomposition. Plastic degradation is rarely complete, as evidenced by a few laboratory experiments. The only part of the plastic materials that is exposed to microorganism activities is the surface. Although microorganisms cannot completely degrade plastics, they do have an effect on the surface properties of plastic materials, such as reducing the size of the material and increasing the total surface area. More contact areas with microorganisms will increase the likelihood of decomposition. Nonetheless, this would lead to the accumulation of plastic particle pollutants, causing competition with microbial activities and the possibility of becoming toxic particles due to chemical absorption [42,44,45].

Normally, plastic biodegradation will involve chemical, mechanical, and thermal degradation. The first step is to increase the surface area of plastic materials before proceeding to the next stage, which involves microorganisms, known as biological degradation. Because of the activities of microorganisms, the products of polymers (plastics) are converted to CO_2_ or CH_4_, water, and biomass [45]. 

Biodegradation in the soil also depends on the general factors mentioned by [40] and [42]. There will be no degradation under poor conditions. However, some of these factors, such as temperature and soil moisture, will prevent plastics from degrading. Temperature has a significant impact on the development of microorganisms, which is one of the primary factors in degradation. The moisture content is important in hydrolytic degradation. It accelerates the decomposition of biodegradable plastics/polymers, such as polylactic acid (PLA). Degradation will not occur smoothly without these two factors being at proper levels. 

**Figure 3:**
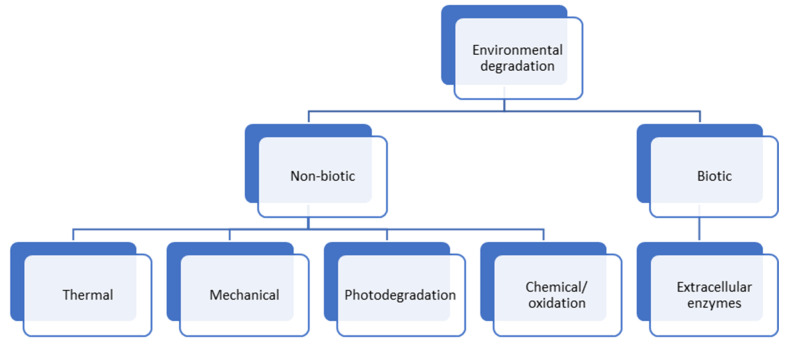
Environmental degradation affects plastic properties.

Plastics in the environment degrade through various processes, such as chemical, thermal, photo, or biological degradation, as shown in Figure 3. Non-biotic activities convert most plastics or polymers into monomers, resulting in a process known as mineralisation. Plastic materials are well known for their complex structure and long carbon chains in their molecules. Microorganisms have difficulty passing large particles through their cell membranes for this reason. Before degradation, plastics undergo physical changes, such as heating and cooling, which would result in structural damage, such as cracking [46]. Then, the small monomers can be absorbed into the cells of microorganisms. In summary, the microbial decomposition of certain plastics may or may not occur without environmental weathering. Biodegradation procedures are limited to observing visual and physical changes.

It also covers polymer molar mass weight loss, CO_2_ evolution and O_2_ consumption, composting, and enzymatic decomposition [47]. The ASTM D5338-92, ISO/CD 14855, and the modified Sturm test ASTM D5209 are all standard methods for testing the biodegradation rate of a material [48]. As a result, understanding the methods that influence plastic degradability is critical for the decomposition process.

## 5. The Potential of Earthworms in Plastics Degradation 

### 5.1. Are Earthworms Widely Used? 

Earthworms are widely used in composting and solid waste management (specifically organic matter, such as food and farm waste). However, in plastic or microplastic degradation, the usage of earthworms is limited. The most commonly used earthworms in plastic and microplastic degradation are *Eisenia Andrei*, *Eisenia Foetida*, and *Lumbricus terrestris.* These earthworms are epigeic types; they prefer to live in compost or leaf litter, rather than mineral soils, and different earthworm species prefer different organic wastes. Even though these earthworm types are widely used in vermicomposting, they are topsoil feeders and have the possibility of early exposure to plastics and microplastics in the soil. Regardless, there are hundreds of earthworm species left to be explored. Limiting plastics and microplastics exposure to only these few species will result in a vague understanding of the capability of earthworms to break down plastic and microplastics, as each species has its own characteristics. However, their ability to break down plastics may differ from anecic and endogeic worms, as their environments or physical features are different. Thus, there has not been enough research utilising enough varieties of earthworm species and their interaction with microplastic in the soil profile. 

### 5.2. Why Do We Think That Worms Should Be Used? 

Earthworms are terrestrial invertebrates. Their digestive system is unique in the sense that it runs throughout their body and a large number of chemoreceptors are located near the mouth. The gut is lined with circumferential and longitudinal muscles that move the digesting food toward the worm’s anus. The worm can move by using similar muscles on the periphery of each segment [49]. Earthworms have the possibility to act as a grinding machine that can physically change the size of microplastics through their muscular digestive tube, with a high surface area for microbial and enzymatic activities. Furthermore, the ability of earthworms to mineralise complex minerals and organic matter affords them a high potential to reduce the degradation time by providing optimal conditions for microbial activity that may break down microplastics into a simple form.

The presence of earthworms in soil will increase the microbial population [50]. Their activities increase the humidity of organic matter and the microbial population, enhancing the presence of auxins and gibberellin-like substances [51]. Earthworms also play an important role in organic matter degradation and soil metabolism through feeding, fragmentation, aeration, turnover, and dispersion [52]. Earthworms have a significant impact on the biogeochemical characteristics of soil. Through feeding, casting, and digging, they contribute to soil physicochemical properties and the microbial grouping [53]. In addition, earthworms provide ecosystem services through pedogenesis, soil structure development, nutrient cycling, water regulation, primary production, pollution remediation, climate regulation, and cultural services. As they eat, they dig holes, turn over the soil, and maintain the substrate like a sponge in an aerobic environment, ensuring oxygen entry and releasing carbon dioxide. 

When they move through waste, they cover their burrows’ surfaces with gelatinous mucoproteins, increasing bacterial activity and subsequent damage. In addition, they cover the top of the bed with a cast that reduces the presence of unwanted odours and flies. This behaviour shows that earthworms physically alter the organic matter and soil and chemically by the mucous on their bodies’ surfaces and their digestive system enzymes and microbes. This may contribute directly to microplastic degradation by the intake of microplastics or through the body mucous indirectly as the earthworm passes by the microplastics on the topsoil or in the burrow walls. 

Their grinding gizzard softens organic material, increases the area of exposure, and expands the beneficial effects of the microbes. Earthworms can extract all of the nutrients from microbes. Microorganisms released from the intestine continue to function temporarily outside of the intestine due to increased peritrophic membranes produced by polysaccharides–mucoproteins. Each peritrophic membrane has amphipathic water properties, with excellent water retention capacity for protection against dryness. The final product retains its shape and structure in the soil, ensuring that food is released slowly without loss, drainage, or immersion. Earthworms can mix various nutrients, allowing them to combine nutrients that provide better nutrition than other forms of fertilisers. In this process, worms worldwide produce substances essential for the biochemistry and functional systems of soils, including enzymes, antibiotics, vitamins, hormones, and trace elements that are very valuable in their digestive system [8,10,54,55,56,57]. 

Despite the fact that microbial activities degrade organic matter, earthworms have an effect on decomposition, either directly by food processing and microorganisms together, or indirectly by reducing microbial populations [58]. By creating optimal habitats for microbial proliferation, earthworms can increase the biodegradable polymer biodegradation rates [59]. This may contribute to the degradation of plastics, as it increases the exposure of the microplastic particles to various microorganisms. Earthworms play a leading role in improving soil conditions by simply swallowing material and discharging organic material. This process can grind plastic materials to a smaller size and cover them with microbial litter, increasing the chance of plastic material degradation. It should be kept in mind that the organic material consumed by earthworms has a high impact on gut microbes. Therefore, soil earthworms may be a suitable solution for microplastics in soil due to their capability to improve soil conditions. 

### 5.3. What Can Be Done to Enable Earthworms to Break down Plastics?

A new degradation procedure should be adapted for plastic degradation that not only considers breaking the plastic down into smaller sizes, but also the chemical changes to the plastic material that may occur due to the degradation process. For example, different types of biodegradable plastics, such as oxo-biodegradable, compostable, and high-density polyethene, did not show any significant indication of degradation within three years of exposure to different environmental weathering processes [36]. Additionally, biodegradable plastics (e.g., cellulose, starch, and polylactic acid) are often mixed with non-degradable plastics. Thus, the degradation of such bioplastics leaves small, non-degradable plastic in the environment, contributing to further problems. Even if the plastic material is crumbled or broken down into smaller sizes, it does not mean that it is degraded. This leads to more complicated problems, such as microplastics and smaller particles accumulating in the environment. There is a limited possibility that these plastics particles may be degraded by the microorganisms as a result of their fine size 

The only solution to the plastic problem is the use of environmentally friendly plastic. In other words, a plastic set that can be converted completely chemically into CO_2_, water, and energy, without any residue in the environment. In addition, exposing the plastic to pre-treatment before exposure to earthworms, such as industrial composting, which is highly recommended for industrial waste materials, provides a suitable condition to enable the breakdown of the plastic materials. This process may weaken the tensile strength of the plastic material and make it more vulnerable to degradation.

## 6. Conclusions and Suggestions

The terrestrial ecosystem has received increasing amounts of biodegradable and non-biodegradable plastic debris and microplastics. However, based on current studies, the biodegradation rate of these biodegradable plastics under natural conditions is not as high as that projected by regulated laboratory testing. Thus, further research is required to manage this plastic pollution. This article reviewed existing studies and, with critical analysis, revealed that earthworms can play a significant role in the biodegradation of plastic and explained the challenges in the process. In addition, this review indicated that earthworms can significantly affect the physical characteristics of plastics through ingestion and mass reduction and can contribute to further degradation. Furthermore, earthworms can play a role as an agent for testing the side effects of these particles in the soil ecosystem.

Additionally, this review discussed earthworms’ abilities to provide a suitable environment for the biodegradation of microplastics throughout their digestive system. Their digestive system provides the correct conditions for a microbial population to flourish by increasing the surface area of the microplastics for further degradation by microbial activity after excreting them through their vermicasts. Moreover, the large variety of earthworms indicates that additional features can be utilised for microbial plastic degradation, as earthworms’ characteristics change based on their territories. Thus, this study suggests a positive relationship between earthworms and plastic degradation. However, there is still a lack of evidence of earthworms’ abilities to chemically degrade or change the chemical structure of plastic and microplastic. Hence, further investigation is required. This study also proposes that sufficient varieties of earthworm species with different environmental conditions must be investigated to determine the possibility of earthworms’ abilities to degrade plastic.

In terms of adaptation, the possibility of earthworms to avoid soil contaminated with microplastics or plastics was observed. Selective feeding behaviour, avoidance of the area containing a high concentration of plastic, and rejection of plastic by earthworms were observed due to their morphological characteristics and because they cause physical damage. Nevertheless, some researchers provided evidence that earthworms can exist in soil containing a certain concentration of microplastics under controlled conditions. Thus, the capability of earthworms to adapt to microplastics in the soil environment is questionable. Again, further research under natural conditions is required to test earthworms’ abilities to coexist and degrade plastics in the natural environment. However, the challenges faced in using earthworms for plastic degradation may be mostly related to the factors affecting the process. The toxicity and complexity of the plastic material, environmental factors, such as temperature and moisture content of the soil, microbial population, and feeding method showed the potential to significantly affect the biodegradation of plastics by earthworms. As a result, pre-treatment is suggested before earthworms are introduced to microplastics or plastic materials. Additional studies should be conducted to understand these factors in order to find a suitable overall condition that can be achieved in the natural environment for the successful implication of earthworms in the degradation of plastics.

The implication of this process is expected to come with side effects, such as the transportation of microplastics into the soil profile and groundwater. Furthermore, it may even contribute to microplastic absorption by plants’ roots due to the earthworms grinding the microplastics into smaller sizes, which can not only cause groundwater pollution, but also damage to plants and human health by entering the food chain. Thus, additional research is required to manage this problem. Earthworms can potentially be used to understand the effect of microplastics on the soil biota and to enhance microbial activities that increase plastic and microplastic degradation. Nevertheless, based on this review, earthworms have a high potential to be utilised as a major contributor to microplastics and plastics degradation. They may be one of the future methods to detect the effect of microplastics on the soil biota and serve as a biodegrading agent.

## Figures and Tables

**Table 1:** Types of studies on earthworms and microplastics.

Type of Study and Finding	Types of Earthworms	Plastic Types	Exposure Condition	References
Fitness and survival of earthworms. At low microplastic concentrations (≤0.5% (*w*/*w*)), no effect was observed. However, significantly inhibited growth and increased mortality of earthworms were detected at higher concentrations (1% and 2%)	*Eisenia Foetida*	Polystyrene	Duration: 30 days Temp: 24 ± 1 °C Moisture: 20% Light/dark: permanent dark Test unit: glass beaker (250 mL)	[22]
Fitness and survival of worms, transporting microplastics to burrows, and degrading microplastics using bacteria from the worms’ guts	*Lumbricus terrestris*	Low-density polyethylene microplastics	Duration: 14 days Temp: 16–18 °C Media moisture: 20% Light/dark: 8:16 h Testing unit: container (300 mm, 405 mm, and 30 mm)	[16,20,21]
Decomposition of plastics. Polymers did not have a toxic effect on earthworms.	*Eisenia Foetida,* and *Eisenia Andrei*	Polyethene, nylon, polypropylene (PP), ethylene vinyl acetate (EVA), and linear low-density polyethene (L-LDPE)	Duration:12 days Light/dark: permanent dark Test unit: plastic container (scale)	[18]
Ingesting plastics (selective behaviour) and transporting microplastics to the soil	*Lumbricus Terrestris*	Polyethene mulch, PLA/PHA, Organix, Bio Agri, Nature cycle, and Weed Guard Plus.	Duration: 50 days Temp: 16 °C Media moisture: 25% Light/dark: 12:12 h Test unit: glass terrarium (30 cm, 30 cm, 4 cm)	[23]
Microplastics are transported into the soil.	*Lumbricus terrestris*	Polyethene microplastics	Duration: 21 days Temp: 20 ± 2 °C Media moisture: 100 mL of water/2 days Test unit: plant pots (3 L)	[15]
Fitness and survival of worms. Both types of plastic negatively affected wheat in terms of vegetative and reproductive growth. However, the presence of earthworms had an overall positive effect on wheat growth and mainly alleviated the impairments caused by plastic residues.	*Lumbricus Terrestris*	Macro and micro sizes Low-density polyethylene and starch-based biodegradable plastic (Bio)	Duration: 129 days Temp: 22–17 °C Media moisture: 70% Light/dark: 14:10 h Test unit: plant pots (2 L)	[24]
Fitness and survival of earthworm. No significant effects were recorded on survival, the number of juveniles, and the final weight of the adult earthworms after 28 d of exposure to different concentrations of microplastics. Histopathological analysis and FTIR-ATR of earthworms’ guts provided evidence of damage and immune system responses to microplastics.	*Eisenia andrei*	Low-density polyethylene microplastics	Duration: 28 and 56 days Temp: 20 ± 2 °C Media moisture: 40% Light/dark: 16:8 h Test unit: polypropylene pots (1500 mL)	[25]
Fitness and bioaccumulation in earthworms. Under environmentally relevant conditions, microplastics should not cause significant toxic effects or enhance hydrophobic contaminant accumulation.	*Eisenia fetida*	Polyethene and polystyrene	Duration: 14 days to 28 days Temp: 25 °C Media moisture: 40% Light/dark: 16:8 h Test unit: glass beaker	[26]
Toxicity—microplastics addition had no significant negative effect on wheat biomass production, wheat seedling emergence, earthworm mortality, growth, or avoidance behaviour, and nematode mortality or reproduction compared with controls	*Eisenia fetida*	High-density polyethene (H-DPE), polyethene terephthalate (PET), polyvinyl Chloride (PVC) microplastics	Duration: 9 months Temp: 23 ± 2.8 °C Media moisture: 60% Light/dark: permanent dark Test unit: borosilicate glass jar	[27]
Ecotoxicity. No mortality. The catalase activity and malondialdehyde content increased significantly at the 1.0 g/kg LDPE concentration after exposure for 28 days. In addition, acetylcholine esterase was greatly stimulated at 1.5 and 1.0 g/kg concentrations of LDPE on days 21 and 28, respectively.	*Eisenia fetida*	Low-density polyethene (L-DPE)	Duration: 28 days Temp: 20 ± 2 °C Media moisture: 20% to 40 % Light/dark: 8:16 h Test unit: glass boxes (15 cm, 5 cm, and 10 cm)	[28]
Transport of the microplastics to lower soil. The biogenic activities of earthworms are a potential pathway for microplastics to be transported into soil and groundwater.	*Lumbricus terrestris*	Polyethene	Duration: 14 days Temp: 16 ± 1 °C Media moisture: 40 ± 5 % Test unit: soil column	[29]
Eco-toxicological soil quality and organisms were not affected at remarkably high doses.	*Eisenia Andrei*	Mater-BI (DF04A, EF04P, AF05S0) (corn starch, bio-based aliphatic polyester, bio- based aliphatic–aromatic co-polyester, natural plasticizers)	Duration: 28 and 56 days Temp: 20 ± 1 °C Light/dark: 16:8 h Test unit: incubator	[30]
The ingestion of polylactic acids can only occur after hydrolytic degradation.	*Eisenia Andrei*	Polylactic acid (50/50 and 96/4 L-lactic- co-D-lactic acids)	Duration: 14–62 Temp: 20 °C Light/dark: permanent dark	[17]
The intake of PLA by earthworms not only stimulates environmental degradation, but also increases the ecological risk caused by nanoparticles	*Eisenia fetida*	Polyethene terephthalate (PET) and polylactic acid (PLA) microplastics	Duration: 10 days Temp: 20 ± 1 °C Light/dark: permanent dark Test unit: testing chamber and 2 L glass beaker	[31]

## Data Availability

All data generated or analysed during this study are included in this review article.
